# The Acoel nervous system: morphology and development

**DOI:** 10.1186/s13064-024-00187-1

**Published:** 2024-06-21

**Authors:** Pedro Martinez, Xavier Bailly, Simon G. Sprecher, Volker Hartenstein

**Affiliations:** 1 Departament de Genètica, Microbiologia I Estadística, Universitat de Barcelona, Av. Diagonal 643, Barcelona, 08028 Spain; 2 ICREA (Institut Català de Recerca I Estudis Avancats), Barcelona, Spain; 3 Station Biologique de Roscoff, Multicellular Marine Models (M3) Team, FR2424, CNRS / Sorbonne Université - Place Georges Teissier, Roscoff, 29680 France; 4 Department of Biology, University of Fribourg, 10, Ch. Du Musée, Fribourg, 1700 Switzerland; 5 Department of Molecular, Cell and Developmental Biology, University of California, Los Angeles (UCLA), Los Angeles, CA USA

## Abstract

Acoel flatworms have played a relevant role in classical (and current) discussions on the evolutionary origin of bilaterian animals. This is mostly derived from the apparent simplicity of their body architectures. This tenet has been challenged over the last couple of decades, mostly because detailed studies of their morphology and the introduction of multiple genomic technologies have unveiled a complexity of cell types, tissular arrangements and patterning mechanisms that were hidden below this 'superficial' simplicity. One tissue that has received a particular attention has been the nervous system (NS). The combination of ultrastructural and single cell methodologies has revealed unique cellular diversity and developmental trajectories for most of their neurons and associated sensory systems. Moreover, the great diversity in NS architectures shown by different acoels offers us with a unique group of animals where to study key aspects of neurogenesis and diversification od neural systems over evolutionary time.

In this review we revisit some recent developments in the characterization of the acoel nervous system structure and the regulatory mechanisms that contribute to their embryological development. We end up by suggesting some promising avenues to better understand how this tissue is organized in its finest cellular details and how to achieve a deeper knowledge of the functional roles that genes and gene networks play in its construction.

## The Acoela and its phylogenetic affinities

The Acoela is an order of small invertebrates that comprises about 400 species, predominantly marine animals that live in a great variety of habitats on Earth [2, 33]. With the clades Nemertodermatida and Xenoturbellida they form the monophyletic group (range phylum), Xenacoelomorpha. This is a phylum of still uncertain phylogenetic affinities. While some authors affirm that xenacoelomorphs are the sister group of the remaining Bilateria (e.g. [10]), others claim an affinity with Ambulacraria (Hemichordata plus Echinodermata) and are, thus, a group of deuterostomes (e.g. [47]). These alternative positions have had, and still have, an impact on our models of ancestral bilaterians, an ongoing discussion that will not be considered any further in this review.

**Fig. 1:**
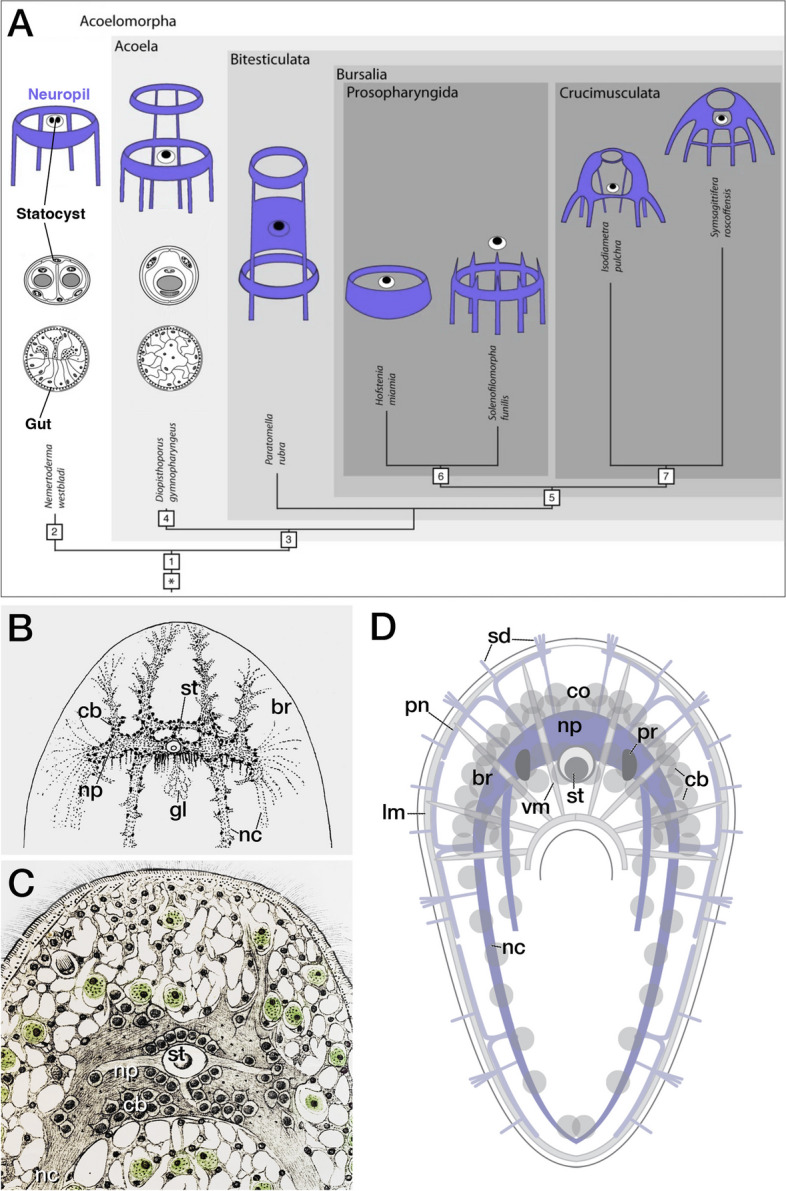
Structure of the acoel nervous system. **A** Phylogenetic context. The acoelomorph ancestor (1) likely had a ring-shaped basi-epidermal neuropil from which a small number of posteriorly directed nerve cords emanated. It possessed a statocyst and a cellular gut. (2) Nemertodermatids retain these attributes; the statocyst acquires two statoliths. (3) The ancestral acoel also displayed one or more ring-shaped (“commissural”) neuropils with multiple nerve cords. Neuropil and cords moved subepidermally. The statocyst retained a single statolith from the ancestral condition; the digestive system changed towards mesenchymal (unpolarized) cells. (4) Posterior pharynx and central digestive syncytium in the most basally branching Diopisthopora. (5) Female accessory organs define the superclade Bursalia. (6) The clade Prosopharyngida acquires a subterminal anterior pharynx. Nerve cords and subepidermal neuropil is lost in clade Hofsteniidae. (7) The ring-shaped pattern of the neuropil is abandoned in favor of a bilobed brains with one to three commissures. Modified from Achatz and Martinez, [1] (with permission). **B**, **C** Structure of the bilobed brain of *Childia groenlandica* based on histological preparations (B, from Westblad, [65]) and reconstruction of the anterior nervous system in *Symsagittifera roscoffensis* from histological observations (C, from Delage, [14]; neuronal nuclei in black, around the statocyst (st)). **D** Schematic representation of a generic acoel nervous system, featuring brain neuropil (np) and cortex with cell bodies (cb), a series of nerve cords (nc) and a peripheral net (pn). Subepidermal meshwork of longitudinal muscle fibers (lm) and transverse/diagonal muscle fibers (not shown). Vertical muscle fibers (vm) between dorsal and ventral surface, in part penetrating the brain. Median statocyst (st) and paired photoreceptors (pr) embedded in neuropil. Ciliated dendritic endings of sensory neurons (sd)

The internal phylogeny of the Acoela has been described in the cladistic study of [32]. In this thorough analysis the Diopisthoporidae is the most basal group within the Acoela (though other uncharacterized, deep-sea, species might be older; see [3]). After the Diopisthoporidae, other clades that diversified, in this order, were the Paratomellidae, the Hofsteniidae (in a sisterhood with the Solenofilomorphidae) and the most inclusive clade of acoels containing a great variety of explored model acoels, the Crucimusculata (a group that contains both the Isodiametridae and Convolutidae). This study allowed, based on the distribution of characters, the modelling of the putative morphology of the ancestral acoel, though not much is dedicated to discussing the ancestral NS architecture. These aspects were discussed in Achatz and Martinez [1] and Martinez et al. [39] (see Fig. 1A). In the next paragraphs we revisit some aspects of the morphological diversification of acoel NS architectures. Before ending this section, it is important here to point out the need of anatomical studies that go beyond the utilization of neurotransmitter antibodies, since they present, necessarily, a biased picture of the neuroarchitecture in every species. The incorporation of unbiased systems, such as those provided by the electronic microscope, would allow us to have a more detailed picture of the NS and, thus, might alter the evolutionary picture we have at present. Our current, more comprehensive, analysis of an acoel NS (that of *Symsagittifera roscoffensis*; done by TEM) is presented below ("Morphological analysis" section). This could serve as a good example for future comparative analysis.

## Anatomy and cytology of the mature nervous system

Understanding the cellular composition of the acoel NS relies, presently, on two approaches: structural, using cellular markers as well as electron microscopy to characterize cell types and tissue organization; and single cell *RNAseq* analysis, which provides us with atlases of cell types, based on coincidences in the gene expression patterns resolved at the single cellular level. In the second case, the resolution can be higher (in terms of the number of cell types/subtypes identified), while in the first, the access to topological information is salient. We will in this section provide a summary of pertinent features of the acoel NS, as a framework that will ease the following description of neural development.

### Morphological analysis

Classical histological studies, in addition to immunohistochemical analyses labeling specific neuron subsets recognizable based on their transmitter expression, have elucidated NS structure for most of the acoel clades (Fig. 1B, C). A few species, notably *Isodiametra pulchra*, *Hofstenia miamia*, *Convolutriloba longfissura* and *Symsagittifera roscoffensis*, have been investigated in more detail (incorporating electron microscopic technologies).

But, before proceeding further, we would like to reemphasize (verbatim from [39]) how we define the acoel “brain”:

*“The term “brain” refers to a conglomeration of nerve cells, typically associated with sensory receptors, in the anterior (relative to direction of movement) part of the body. Most depictions of acoelomorph architecture include an anterior brain.*

*It is likely that, in cases that emphasize the absence of a “true brain” (e.g., *[52]*), only specific, sparsely distributed neuron types were labeled, which does not allow one to draw conclusions about the extent and structure of the nervous system. In some papers, the anterior brain is called “cerebral ganglion” or “nerve ring” (nerve ring understood in the literature as a “circular neuropil”). A distinction between “brain” and these other terms lacks clear (*= *quantifiable) criteria. All three terms refer to conglomerations of neuronal cells, associated with a voluminous neuropil formed by neuronal processes (neurites) and synapses, thus the term “brain” is used in the following.”*

In this context and notwithstanding the considerable diversity of NS structure in different clades, the acoel NS has three structural components in common (Fig. 1D):1) a central nervous system that includes an anterior brain plus a series of longitudinal and commissural nerve chords; 2) a peripheral nerve net that extends throughout the whole body; (3) peripheral ciliated endings of sensory neurons that project to the brain and nerve cords. Brain and nerve cords consist of a more or less densely packed layer of cell bodies (cortex or “rind”) surrounding long nerve fibers, terminal branches and synapses, all of which make up the neuropil (Fig. 1D), though in some acoels the relative position of cell bodies and surrounding neuronal processes (or plexus) can be variable ([1] for a discussion). A statocyst is typically embedded in, or close by, the neuropil of the brain. Brain and nerve cords, as well as the peripheral nerve net, do not display an overt dorso-ventral polarity: Nerve cords of similar diameter are distributed evenly around the circumference of the body, whereby dorsal or ventral cords can be more pronounced depending on the specific clade looked at [48].

All the above-mentioned features are already present in members of the Diopisthoporidae, which are considered as the sister taxon to all other acoels and is similarly seen in other taxa that branch off the acoel phylogenetic tree at intermediate and high levels (Fig. 1A). Thus, in *Diophisthoporus psammophilus* and *Diophisthoporus longitubus* the NS was characterized as an anterior neural mass surrounding the statocyst from which six nerve cords extend posteriorly at different dorsoventral positions [15, 64]. These and other authors [36, 49] also document the presence of an extensive peripheral nerve plexus. The shape of the neuropil, in Diopisthoporidae as well as other basal taxa (e.g., Paratomellidae, Prosopharingida, is often described as “commissural”: nerve processes of central neurons are organized in one or more ring-shaped tracts that surround the anterior tip of the body at the level of the centrally located statocyst, and that emit anteriorly and posteriorly directed longitudinal tracts ([1], Fig. 1A). In contrast, the structure of the neuropil in more highly derived clades (Crucimusculata; including the genera Neochildia, Isodiametra, and Symsagittifera; [32]) is characterized as “bilobed”, whereby (possibly because of a higher number and density of neurons) the overall size of the neuropil is increased and has adopted the shape of two lobes enclosing the statocyst (Fig. 1A, B, C, and D).

**Fig. 2:**
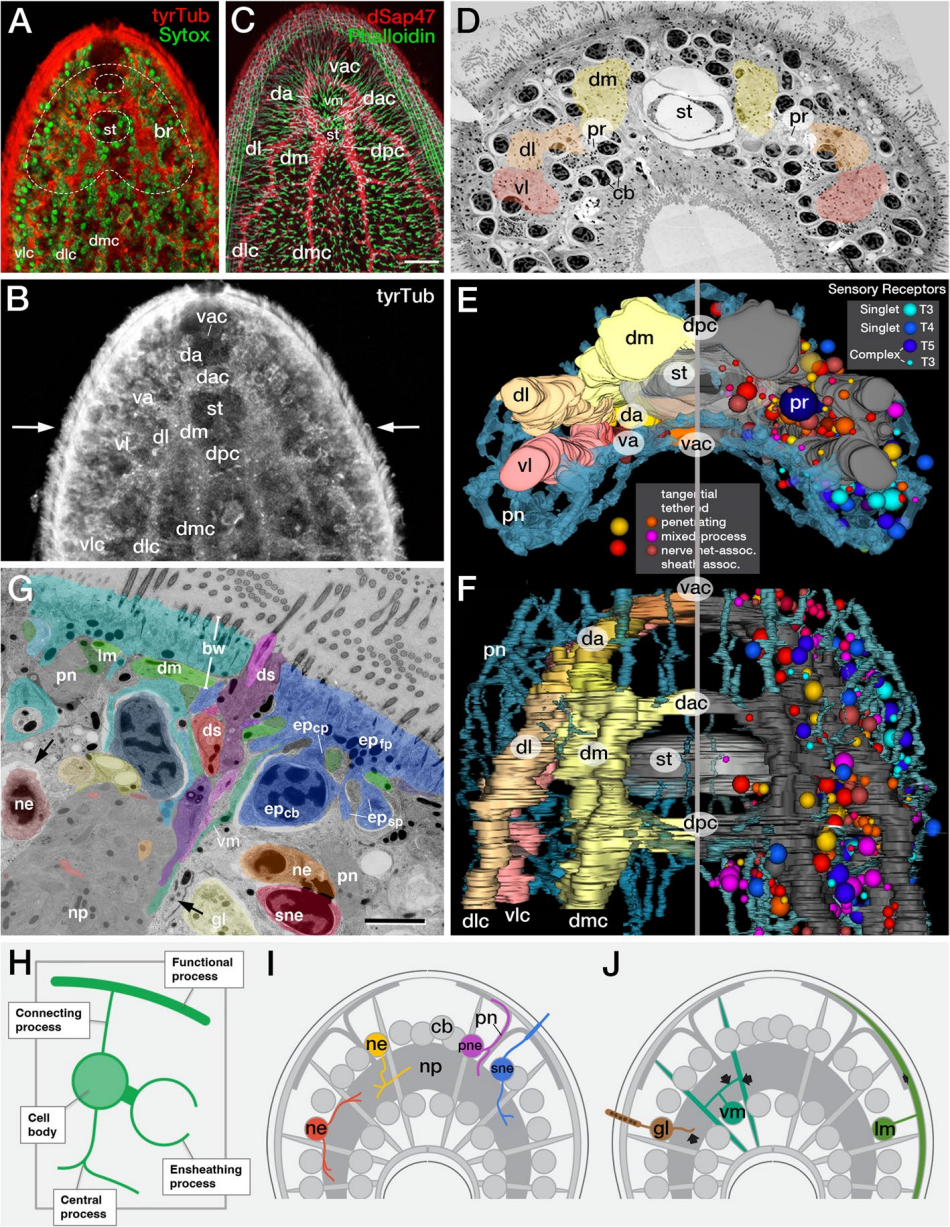
Brain of Symsagittifera roscoffensis (**A-C**) Z-projections of dorsal set of confocal sections of the head of a juvenile (a, b) and adult (c) worm. The nervous system is labeled with anti-Tyrosine tubulin (a, b) and anti-synaptotagmin (dSap47, c); nuclei are labeled with Sytox (a), muscle fibers with Phalloidin (c). The neuropil of the bilobed brain (br; hatched line in A) flanks the statocyst (st) and is comprised of five lobes (da: dorso-anterior lobe; va: ventro-anterior lobe; dm: dorso-medial lobe; dl: dorso-lateral lobe; vl: ventro-lateral lobe) filled with longitudinally oriented fibers, connected by three commissures (vac: ventro-anterior commissure; dac: dorso-anterior commissure; dpc: dorso-posterior commissure). Note**.** The ubiquitous occurrence of densely spaced vertical muscle fibers (vm) penetrating the brain. **D** Electron micrograph of cross section of head of juvenile *S. roscoffensis* at the level of the statocyst (level indicated by arrows in (**B**). The brain neuropil (shaded) is surrounded by cell bodies (cb) of neurons and other cell types and is divided into dorso-medial lobe (dm, yellow), dorso-lateral lobe (dl, orange), and ventro-lateral lobe (vl, red). Embedded in the medial edge of the neuropil, close to the statocyst, are the bilateral photoreceptors (pr). **E**, **F** 3D digital model of brain of a *S. roscoffensis* juvenile in posterior view (**E**) and dorsal view (**F**). Both halves of panels (**E**) and (**F**) feature the same (left) brain hemisphere; right halves are horizontally flipped. On left halves of models, the neuropil lobes (dm, dl, vl) are color-coded as in panel (**D**); on right halves they are rendered in gray. The meshwork of thin anastomosing fiber bundles formed by the peripheral nerve net is rendered in blue in both halves. Right halves of model also display neuronal cell bodies rendered as spheres in different colors (see color keys for central neurons and sensory receptor neurons at bottom and top, respectively, of panel (**E**). Diameter of spheres representing central neurons reflect number of processes emanating from cell bodies (small: one process; medium: two processes; large: three or more processes). **G-J** Structural aspects of neurons and other cell types of *S. roscoffensis* juvenile, based on serial EM analysis. (**G**) shows electron micrograph of cross-section of juvenile *S. roscoffensis*, illustrating structures of the body wall (bw), the cell body domain (brain cortex), and the brain neuropil (np). Different types of cells are rendered in blue (epidermal cells), green (muscle cells), red (neurons) and yellow (gland cells). The epidermal cell at upper right illustrates the “canonical” architecture of acoel cell types, schematically depicted in panel (**H**), consisting of a cell body (ep_cb_) sending out a connecting process (ep_cp_) to the functional process (ep_fp_), in addition to one or multiple sheath processes (ep_sp_). Muscle cell fibers include longitudinal fibers (lm), diagonal fibers (dm) and vertical fibers (vm). Also shown are a bundle of peripheral sensory dendrites (ds; shades of purple), cell bodies of central neurons (ne) and sensory neurons (sne) and processes of glands (gl). These cell types are all intermingled within the layer of somata that surround the neuropil (np). Note profuse ensheathing processes surrounding most somata (black arrows); sheaths are mostly formed by epidermal cells as well as the digestive syncytium. (**I**, **J**) Schematic representations of *S. roscoffensis* juvenile, visualizing the processes of central neurons (ne; tangential neuron: red; penetrating neuron: yellow), peripheral neuron (pne; magenta), sensory neuron (sne; blue), vertical muscle cell (vm; turquois), longitudinal muscle cell (lm; green) and gland cell (gl; brown) in relationship to the neuropil (np), cell body domain (cb) and peripheral nerves (pn)

Shown as an example in Fig. 2 is the brain of a *S. roscoffensis* juvenile. In this species (and similar to what has been described for *Neochildia* [9, 50] and *Isodiametra* [1]) most of the volume of the anterior one third of the juvenile animal consists of a neuropil surrounded by a layer of cell bodies that include neurons, as well as other cell types, such as muscle and gland cells (Fig. 2A, B). The neuropil contains at its center the unpaired statocyst, flanked by a bilateral pair of ocelli (Fig. 2C, D). Muscle fibers and gland necks pass through the brain neuropil. The shape of the neuropil resembles that of a pair of vertical plates linked by three commissures, including a ventral anterior (vac) and dorso-anterior (dac) commissure bordering the statocyst, and a dorso-posterior (dpc) commissure linking the posterior wings of the neuropil ([4, 58]; Fig. 2B, F). Towards posteriorly, the neuropil breaks up into three bilateral symmetric nerve cords [dorsomedial cord (dmc), dorsolateral cord (dlc) and ventrolateral cord (vlc)] that extend all the way into the tail of the animal. The system of commissures provides landmarks to subdivide the neuopil into several discrete domains. Along the anterior–posterior axis, the insertion of the commissures defines a dorsal and ventral anterior neuropil (da, va: between vac and dac commissure), and a dorso-medial, dorso-lateral and ventro-lateral neuropil (in between dac and dpc; Fig. 2B, C, F).

Further analyses of serially sectioned and electron microscopically imaged *S. roscoffensis* juveniles (B. Gavilan, PM, VH, SSp) can shed light on the fine structural details of how neurons and associated cells, such as sensory receptors, muscles or glands are organized in the NS, and that is what we have done recently using serial TEM. Briefly, our new (comprehensive) dataset was generated in the following way. A hatchling specimen of *S. roscoffensis* was included in epoxy resin and cut in 941 Sects. (70 nm each), representing the most anterior part of the animal.100 pictures were taken per section, stitched and aligned yielding about 94,000 pictures to be analyzed using TrakEM2, an ImageJ software plugin. Cell profiles were followed through the stack and their morphology reconstructed. The identities of these cells were assigned/determined manually. A, final, 3D map of cell types with their spatial locations in the body is in the process of completion.” Compared to other bilaterian taxa, acoels exhibit several peculiar morphological features, correlated with (and possibly caused by) the absence of basement membranes. An important role of basement membranes is to compartmentalize organs and tissue layers, such as epidermis, musculature, nervous system, and digestive tract. Enclosure by basement membranes endows cells with a regular packing and more or less isomorphic shapes (e.g., cuboidal, cylindric, fibrous). In the acoel, lacking basement membranes, cells can extend processes and intermingle (enwrap) with each other within the interior of the animal (“parenchyma”). Most cells have uncommon shapes whereby the cell body, formed by a nucleus surrounded by a thin cytoplasmic fringe, is separated from the “functional compartment” of the cell (e.g., the contractile muscle fiber, epidermal sheath, gland neck) by long “connecting processes” (Fig. 2G, H). Furthermore, many cells, and in particular the digestive syncytium that fills much of the body cavity, extend abundant sheath-like processes that wrap around cell bodies (Fig. 2G).

Based on the architecture of their processes and relationship to neuropil, nerve net and epidermal layer, neurons can be classified into central neurons (processes confined to neuropil), nerve net-associated neurons (at least one process into nerve net) and ciliated sensory receptor neurons (dendritic processes penetrating epidermal sheath; Fig. 2E, F, I). Irrespective of these classes, all neurons have cell bodies that lie side by side within the cortex around the neuropil, with sensory receptor neurons more densely populating the ventro-lateral fringes of the cortex, and nerve-net associated neurons typically situated alongside fascicles of the nerve net penetrating the cortex (Fig. 2E, F). As stated above, neuronal cell bodies are often framed by thin ensheathing processes that originating from adjacent cells, be the other neurons, muscle cells, epidermal cells, or the digestive syncytium (Fig. 2G). The majority of central neurons are unipolar or bipolar (details on neuronal morphologies will be described in: VH, PM et al. in preparation), with processes extending along the surface of the neuropil before sending short terminal branches towards the neuropil center (“tangential neurons”; Fig. 2E, F, I). Other neurons have one or two processes that (orthogonally to the surface) penetrate the neuropil (“penetrating neurons”; Fig. 2E, F, I). Neurons with processes, typically two or three, combining these properties (“mixed process neurons”; Fig. 2E, F) also occur. A surprisingly large number of neurons, independent of number and shape of their processes, are filled with dense core vesicles, indicating that they may interact by peptidergic neurotransmission [4]. Sensory neurons are typically bipolar, with one process (dendrite) protruding peripherally and ending in between epidermal cells as one of the three types of sensory receptors observed in *S. roscoffensis*, including non-collared [type 3 (T3)] and collared [type 4 (T4), type 5 (T5)] receptors [4, 63]. Sensory axons typically proceed as thin bundles that directly project to the neuropil of the brain and longitudinal trunks, without contributing to the peripheral nerve net (Fig. 2G, I). This net is formed mainly by processes of “nerve net-associated” neurons (Fig. 2E, F, I). The branching pattern of these nerve net-associated neurons, as well as their endings in relationship to other cells (e.g., muscles, sensory receptors), has not been elucidated yet.

**Fig. 3:**
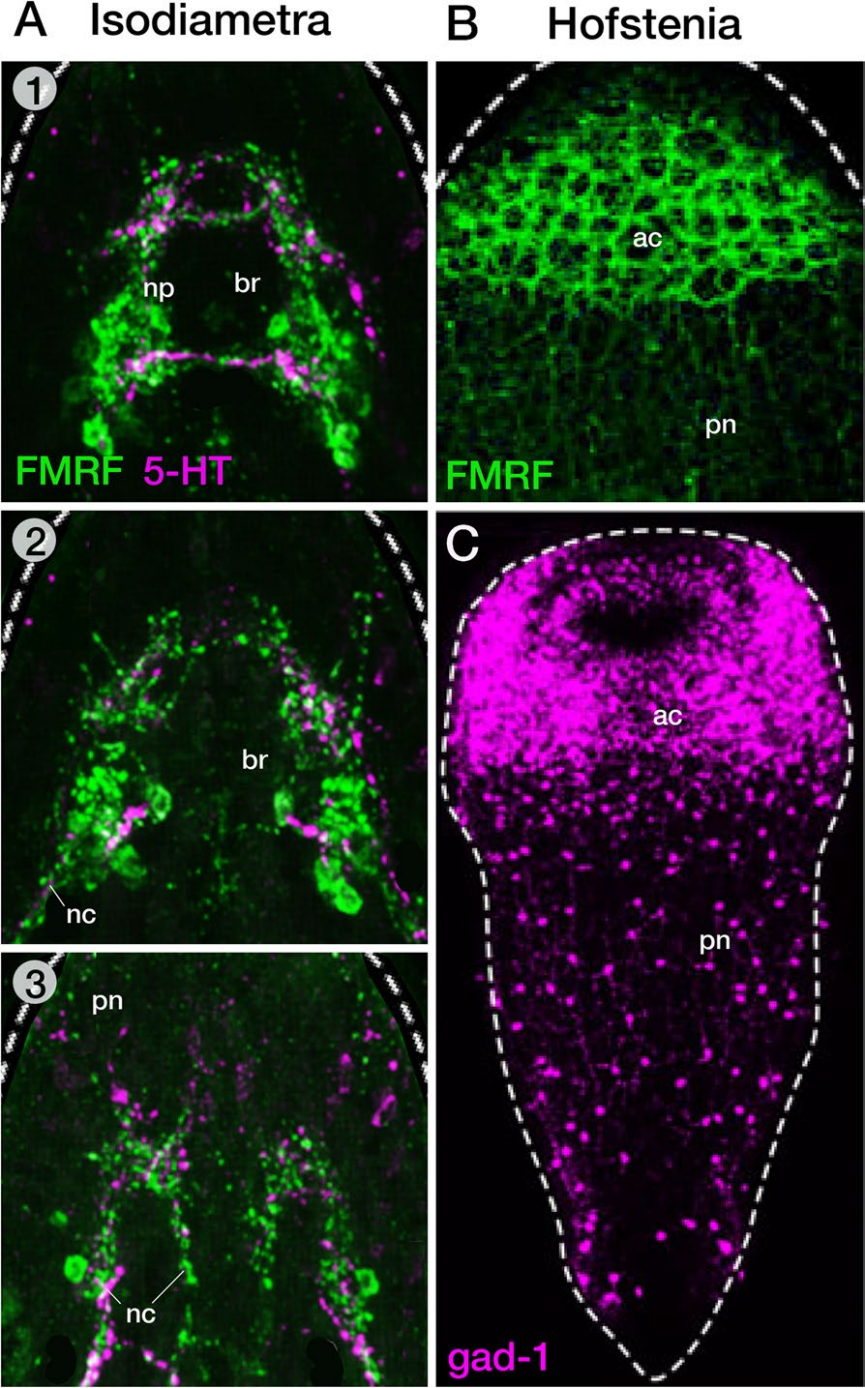
Nervous system of *Hofstenia miamia* and *Isodiametra pulchra. *In *Isodiametra* (**A**; 1-3 show z projections at different dorso-ventral levels), labeling with FMRFamide (green) and 5-HT (magenta) reveals a bilobed neuropil (np) in the center of the brain (br), and longitudinal nerve cords (nc) projecting posteriorly. In *Hofstenia* (**B**), FMRFamide-immunoreactivity appears as a subepidermal network in the anterior part of the body (anterior condensation, ac). Nerve cords are absent. The expression of gad-1, also in Hofstenia (**C**) shows a similar pattern of peripheral neurons, scattered loosely throughout the trunk, and packed densely in the anterior condensation (ac). Images from Hofstenia are taken from Hulett et al., [29], with permission. The images from Isodiametra are taken from Achatz and Martinez, [1]

We would like to end this brief survey of acoel neuroanatomy by drawing attention to the special case of the taxon Hofsteniidae, member of the basally branching Prosopharyngidae [32]. *H. miamia* has evolved as one of the experimentally amenable and heavily studied acoel models [57], and many details of neural structure and development have emerged in recent years. As opposed to the bilobed brain emitting nerve cords shown by other taxa (e.g., *Isodiametra*, Fig. 3A) the nervous system of *Hofstenia* appears as an “anterior condensation” of a large number of fairly peripherally located neurons integrated in an extensive peripheral nerve net (Fig. 3B, C). The condensation has the shape of a subepidermal cylinder completely encircling the anterior tip of the body [5, 11, 59]. The cylinder is thickest dorsally, gradually becoming thinner towards the ventral side.

Using many molecular markers, notably markers for transmitters such as FMRF, ACh and GABA/Glutamate, Hulett et al. [29] describe the anterior condensation as being “composed of reticulated neurite bundles, i.e. bundles of neural processes, that are wrapped around clusters of nuclei”. These authors distinguish between two layers, an outer layer (“layer I”) rich in neurite bundles and an internal layer densely packed with neuronal cell bodies (“layer II”). A typical brain neuropil (as characterized in the sections above), or longitudinal nerve cords are absent. The only structure, aside from the extensive nerve net meandering throughout layer I, that might be considered as some sort of neuropil is described as a “dorsal commissure”, a plate of nerve fibers arching over the pharynx [29]. The statocyst is located in the center of the animal, at the level of the dorsal commissure, but otherwise not connected to this commissure or the anterior condensation. That being said, the statocyst is accompanied by a small number of neurons [59]; a structure, though, that is not clearly detected in Hulett et al. [29]. Some early observers of acoel neuroanatomy (e.g., [64] consider this “statocyst ganglion” as the “true” brain of *Hofstenia*, while interpreting the superficial anterior condensation as a derived specialization of the nerve net that is present in all acoels. Other authors (e.g., [59]), on the other side, take the peripheral nerve net (rather than a brain with longitudinal cords) as a primitive feature. According to this author, *Hofstenia* could represent an intermediate stage between animals that only have a peripheral plexus (e.g., cnidaria) and the “higher” acoels (e.g., Crucimusculata) with a definitive brain and longitudinal cords. However, this view is contradicted by the presence of a brain/nerve cords already in Diopisthoporidae or other Prosopharyngida (e.g., Solenofilomorphidae), and we have to therefore consider the peculiar neuroanatomy of *Hofstenia* as a secondarily derived trait.

### RNAseq based clustering

A few studies have been devoted to the characterization of the cellular composition in acoels. In the case of single cell sequencing, those for *Isodiametra* (hatchlings) and *Hofstenia* (different postembryonic stages; from hatchlings to sexually mature adults) deserve mentioning [16, 35]. In all cases, the analysis of cell clusters has uncovered the presence of a variety of cell types, including neurons. Duruz and collaborators identified 12 neuronal subclusters, which were classified according to the expression of specific markers. Specifically, these clusters could be assigned to the following neuronal types: immature/differentiated neurons (expressing growth factor, mitosis, and DNA synthesis genes), cholinergic neurons (choline acetyltransferase) and sensory neurons (expressing transient channel potential genes, TRPs, and thus called TRP + neurons).

Interestingly, among the TRP + cells, a fraction contains opsins and are considered likely to be photoreceptors (some of these are in the periphery of the brain). Other TPR + cells are in the anterior tip of the animal and, also, in the periphery of the brain. Serotonergic neurons were also identified using serotonin transporter genes. They are in the anterior area of the brain, neuropil, and cell bodies, and are likely ciliated (given the expression of microtubule stabilizing gene products). The complementary use of specific antibodies suggests the bipolar morphology of those serotonergic cells. The location of these cells is consistent with previous published results [1]. Four additional clusters were identified as chemosensory (use of different sets of sodium channels), some of which expressed glutamate receptors and, others, receptors for acetylcholine. The location of these cells is also in the area of the brain potentially devoted to sensing environmental compounds. A cluster of cells expressing several lipoprotein receptors has been identified and tentatively assigned a support (metabolic) function in the brain. Neuropeptides, well identified in the *I. pulchra* genome [62], were expressed in different cell clusters, but these included both neuronal and non-neuronal ones (probably secretory), thus making it difficult to clearly identify peptidergic neuronal types. Peptidergic neurons have been identified previously in different acoels (including *Isodiametra*) using antibodies and immunochemical methods (e.g., [1, 4, 29, 39, 49, 52, 53, 55]).

In the case of *Hofstenia*, the use of single cell sequencing has also produced an extensive atlas of cell types that include different neuronal subtypes [30]. In this study, however, the focus is on the trajectories and dynamics of stem cell (neoblast) population and, thus, further characterization of neuronal, and other, subtypes are not addressed.

## Structural aspects of nervous system development

### Cell lineages formed during cleavage and gastrulation.

Acoel embryos develop directly into juveniles, without the formation of a larval stage. Since the original work of Georgévitch [22] and Bresslau [8], the studies of cell lineage in acoels have been mostly limited (and with varying success) to a couple of well-described species, namely *N. fusca* [26] and *H. miamia* [34, 35].

**Fig. 4:**
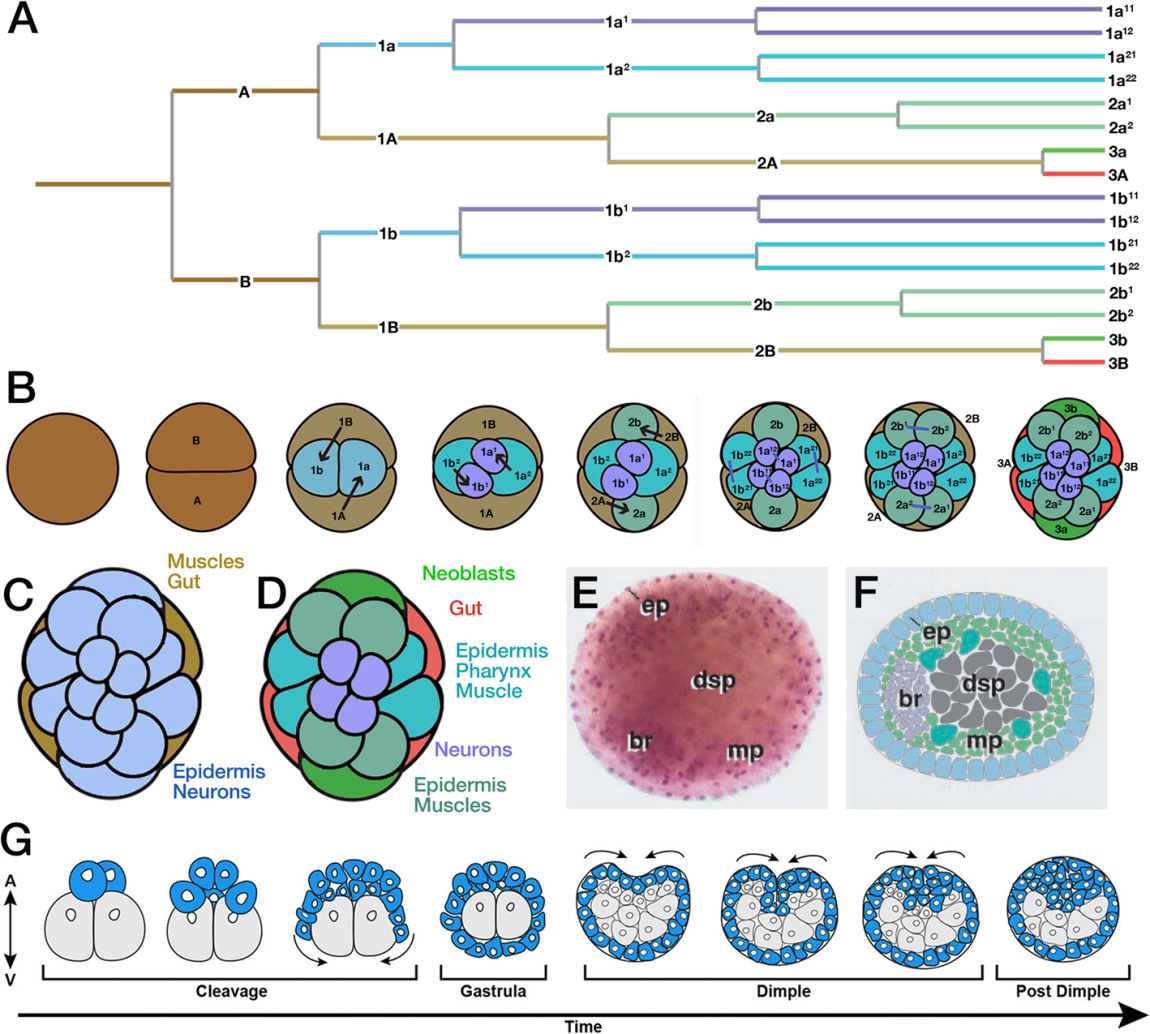
Development of the acoel nervous system. **A**-**D** Duet-spiral cleavage and blastomere fate, based on the analysis of *Neochildia fusca* [26] and *H. miamia* [35]. Panel (**A** depicts sequence of annotated blastomere divisions, with spatial arrangement of blastomeres shown in (**B**, view from animal pole of embryo. Rendering of blastomeres with different colors reflects fate, as mapped for *H. miamia* by Kimura et al. [34, 35]. The association of color and fate is provided in panel (**D**), which shows the 16-cell morula of Hofstenia. Panel (**C**) depicts Neochildia, for which Henry et al. [26] fate mapped all micromeres to epidermis and neurons, and the 3A/3B macromeres to muscle, the statocyst cell producing a statolith and the gut. (**E**, **F**) Appearance of the brain primordium in a stage 5 (48–72 h,corresponds to “post dimple stage) embryo of *N. fusca* (from [50], with permission). (**E**) shows fuchsin-labeled whole-mount (embryo shown in ventral view; anterior to the left); (**F**) represents a schematic drawing of sagittal section. The brain primordium (br) forms a bilaterally symmetric condensation at the anterior pole underneath the epidermal primordium (ep) that at this stage has differentiated into a cuboidal ciliated epithelium. The muscle primordium (mp) forms a thin layer of scattered subepidermal cells. (**G**) Schematic representation illustrating the two cell-internalization events that occur during development of *H. miamia* (adapted from [35], with permission). The first event is gastrulation, whereby, following cleavage, the two vegetal macromeres are overgrown by the micromeres. At a later stage (“dimple stage”), outer cells at the animal pole ingress,these cells, presumably, give rise to neurons, muscle cells, and other internal cell types

Using injection of lineage tracers in different blastomeres of early embryos and following their descendance, these authors have shown the early origin of the major organ systems, including the nervous system, epidermis, musculature, neoblasts, and gut. Acoels exhibit a unique form of cleavage termed “duet spiral cleavage”, whereby the first two divisions generate a pair of small cells (micromeres), 1a/1b at the animal pole, and two large cells (1A/1B; macromeres) at the vegetal pole (Fig. 4A, B). Subsequent divisions generate 3 duets of micromeres (1a1/1b1, 2a/2b, 3a/3b) and 2 macromeres (3A/B), whose further mitotic activity leads up to a blastula of approximately 30 micromeres capping two large macromeres (3A/3B) that later, during gastrulation, become internalized (Fig. 4B, G). In *Neochildia*, the outer, micromere layer forms the ectoderm, and all three micromere quartets generate epidermis and nervous system (Fig. 4C). Macromere duet 3A/3B generates internal tissues, including musculature, the statocyst cell producing a statolith and the (syncytial) gut (Fig. 4C). Interestingly, some experiments in which different blastomeres were deleted show that the specification of micromere fate (including nervous system) seems to be dependent on interactions with the underlying macromeres [7],the embryo shows a classical regulatory nature.

Recently, Kimura and collaborators [35] have used lineage tracing methodologies in the *Hofstenia* embryo (using photoconversion labelling). They injected and followed the destiny of each blastomere at the 8-cell and 14 cell stage embryos. Cells become specified, yet not committed, as neuron, muscle, epidermis, neoblast and gut as early as the 8-cell stage. There are two fundamental differences to the findings by Henry et al. [26] in *Neochildia*. Thus, in *Hofstenia*, only the animal-most micromere duet, 1a1/1b1, produced the nervous system. 1a2/1b2 and 2a/2b generate muscle, epidermis, and pharyngeal tissue, but no neurons (Fig. 4B, D). Micromeres 3a/3b are the progenitors of neoblasts, self-renewing stem cells that post embryonically regenerate all other cell types [30], macromeres 3A/3B produce the statocyst cell producing a statolith, mid-section cells and the gut (Fig. 4D). In other words, different from *Neochildia*, and more in line with other (spiralian) bilaterian taxa, such as annelids, macromeres do not constitute a “mesoendoderm”, but only “endoderm”, in *Hofstenia*.

Even though the different blastomeres of the embryo give rise to different cell types and tissues in an invariant pattern, these cells are not yet committed/locked to their respective fates. As already indicated in classical ablation studies by Boyer et al. [7] and Henry et al. [26] and reinvestigated rigorously by Kimura et al. [34, 35] for progenitors of neurons and neoblasts in *Hofstenia*, ablation of individual blastomeres in the early blastula does not bring about the loss of cell types normally produced by the missing blastomeres. In other words, blastomeres determined to generate a certain type, such as epidermis or muscle, are able to switch to the production of neurons in case that the 1a1/1b1 micromeres, the exclusive source of neurons in normal development, are absent. Gene expression studies strongly supported the ablation studies, showing that cognates of genes associated with the specification of cell fates do not come up in the blastula, but at relatively late (post-dimple) stages when internalized cells start to coalesce into discrete organ primordia. One has to take this finding into account when conceptualizing the mechanism by which the acoel body is patterned: even though, due to the invariant pattern of cleavage division, certain blastomeres can be associated with specific positions in the juvenile body (e.g., the 1a1/1b1 duet at the animal pole with a dorsal position in the juvenile; [35]), the definitive setting of positional information in the body and nervous system, as discussed in the following, is likely to take place at much later stages of embryonic development.

### Neurulation: Internalization of cells and nervous system morphogenesis

Following gastrulation, neural progenitors separate from the ectoderm in a process called neurulation. In Bilateria, including derived clades such as hexapods and chordates, the neurulation process follows a stereotypical sequence of events: (1) A specific domain within the ectoderm (called “neuroectoderm”) undergoes a change in cell shape, with cells adopting an (apico-basally) elongated shape. In chordates, this domain, called neural plate, forms in the dorsal ectoderm and subsequently invaginates as the neural tube. In hexapods, the neurectoderm forms ventrally, and rather than undergoing whole-sale invagination, gives rise to scattered neural progenitors (“neuroblasts”) that individually, or in small groups, delaminate interiorly; (2) Internalized neural progenitors proliferate in complex patterns over a prolonged period to give rise to neural precursors that subsequently emit fibers and differentiate into neurons.

Little is known about neurulation in acoels. The end product of neurulation, the brain primordium, has been described, as it appears around mid-stages of development [stage 5; day 4 in *Neochildia* [50]; Fig. 4E, F); revealed by gene expression at 24 h and unequivocal morphological detection at 5 days in *Symsagittifera* [4, 45], as a bilobulate mass of neuronal precursors that appears at the anterior pole of the body (approx. 250 cells/lobule). Cell differentiation ensues during the fifth day of embryogenesis (stages 7 and 8). The internal neuropil will eventually be crossed by newly specified muscle and glandular cells, giving rise to the characteristic aspect of the acoel nervous system, as described above.

The morphogenetic events of neurulation have been elucidated in good detail for *Hofstenia* in a couple of recent papers [34, 35]. These authors demonstrated that the entirety of cells descending from the most apical micromere duet, 1a1/1b1, segregate from the ectoderm shortly after gastrulation, a stage called “dimple stage” in referring to the characteristic indentation that appears at the animal pole of the embryo (Fig. 4G). During later stages, these internalized cells further multiply and assemble into the “anterior condensation” that constitutes the *Hofstenia* brain. Cells extend processes that grew into the peripheral nerve net. In addition to these neural precursors, part of the progeny of micromere duet 1a2/1b2, as well as all of the descendants of 3a/3b, also internalize during the dimple stage. The former give rise to the musculature, the latter to the neoblasts, a pool of pluripotent progenitors that account for postembryonic growth, cell renewal and regeneration in all acoels [12, 21]. Kimura et al. [35] also analyzed the fate of neoblasts, as it pertains to cell renewal and regeneration, by following labeled 3a/3b descendants into post-hatchling stages, as well as ablating these cells and irradiating animals. The results demonstrate that prior to hatching, and even at least 1 week after hatching, neoblast-derived cells do not become incorporated into the nervous system, indicating that the 1a1/1b1 micromeres and their descendants are the only source of neurons of the juvenile worm. This is important because during adulthood, all neurons that are added to the existing pool, or replace aged neurons, come from neoblasts [35]. In this regard, acoels, along with platyhelminths which possess a similar system of pluripotent neoblasts, present a unique mechanism of cell renewal: instead of delegating cell renewal to specialized stem cell populations housed in the different organs, such as nervous system or epidermis, these animals retain motile, pluripotent stem cells that roam the body and act as a source for all cell types.

## Patterning the acoel NS: molecular aspects of neural development

While NS architectures are known for a group of acoels [39], there is still a limited amount of information on the use of patterning genes to organize it, in time and space. This is, in part, due to the paucity of in situ data produced in recent years, and the practical difficulties of obtaining well-stained embryos, juveniles and adults. Currently, due to the quick generation of transcriptomes in several acoels and better access to probes (plus improved in situ methodologies), we can obtain data on a larger scale. In the field, we still depend on the candidate gene approach, thus the families for which we have better understanding are those that traditionally have been used as markers for different stages of body organization, including neurogenesis, from the initial Antero-Posterior (AP) and Dorso-Ventral (DV) patterning systems to the expression of vectorial systems, such as the Hox, directing the regionalizing the body along the major body axis to members of other transcription factor families, involved in different stages of maturation of the NS. While most of these genes have been analyzed in the context of juveniles, very little is still known of their expression in the embryonic stages and their specific role in the specification and differentiation of neural populations. This lack of proper, and detailed, markers complicates the analysis of putative homologies between nerve chords and brains in the Acoela and other bilaterians [60].

**Fig. 5:**
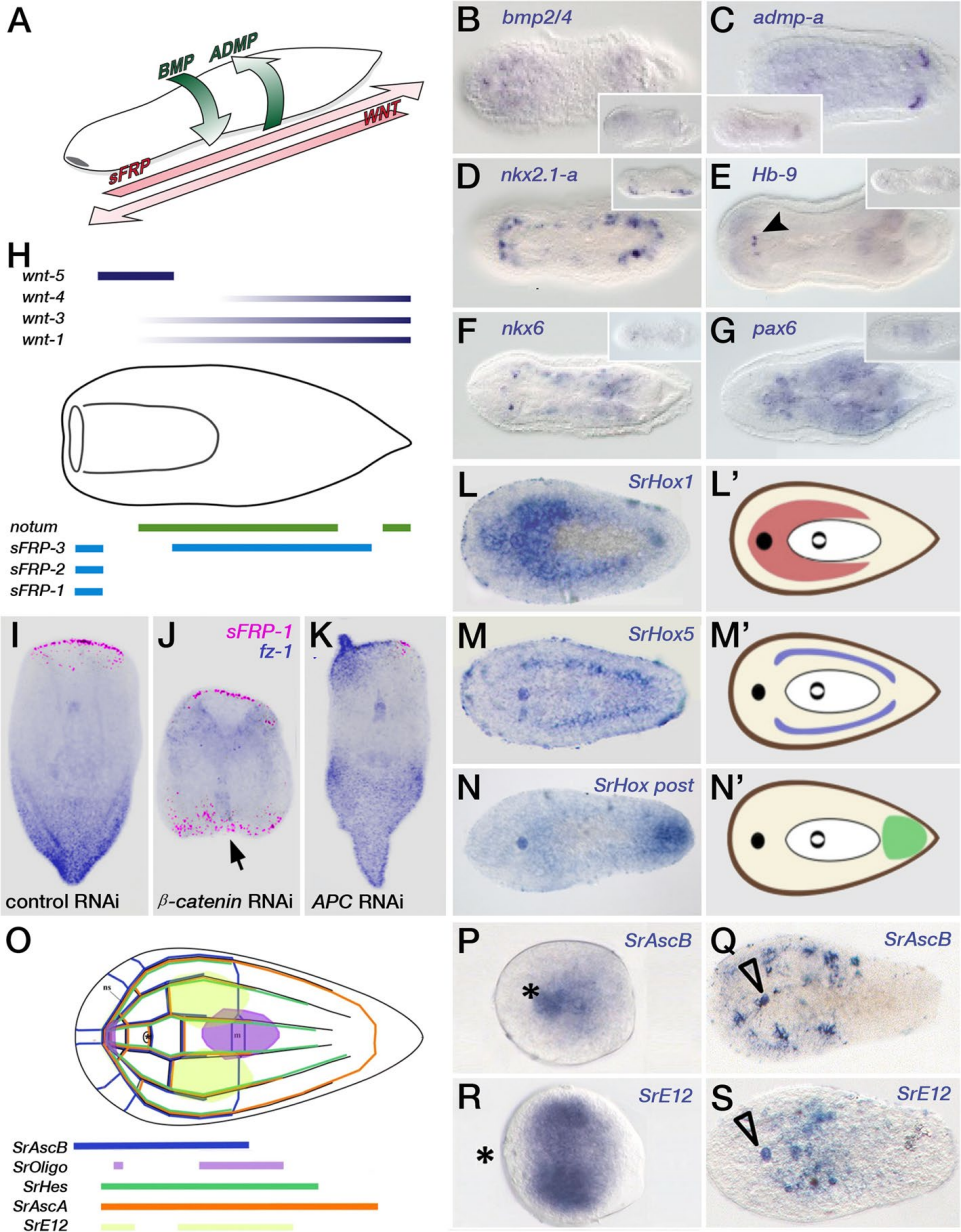
Patterning the acoel nervous system. **A **The diagram represents the A-P and D-V major gene regulators in *H. miamia* [56]. The A-P axis is specified through the interplay of members of the Wnt family of ligands and their antagonists, FRPs (Frizzled Related Protein). The D-V axis, instead, is regulated by the Bone Morphogenetic Pathway, represented here by the opposite actions of BMP ligands and their antagonists (ADMP). **B-G** Gene expression patterns of the D-V specifying pathways (BMP-ADMP,as in panel A), plus their putative downstream effectors, regulating the position and specificity of neural structures along the bilaterian’s D-V axis; all are members of different homeobox-containing families (*nkx2.1-a*, *Hb-9*, *nkx6* and *pax 6*). Data obtained from Martin-Duran et al. [38], with permission, in the species *I. pulchra*. **H** New, detailed, diagram showing the expression domains of members of the Wnt family of ligands and their FRPs antagonists. The scheme follows the simplified one represented in panel A. The position along the A-P axis of the different Wnt and FRP expression domains is slightly different, and in most cases, are overlapping (diagram from [56], with permission). **I-K** Effects of knocking down, using RNAi, the activities of *β-catenin* and *APC* in regenerating specimens of *H. miamia*. β-catenin is a downstream effector of the Wnt signaling pathway while APC (Adenomatous Polyposis Coli), is a negative regulator of the β-catenin concentration (**I-K**) Functional analysis of some A-P gene regulators in regenerating fragments of *H. miamia*. **I** Expression of the anterior marker, *sFRP-1,* after the inhibition of a control gene (a fragment of the C. *elegans unc-22*). *Hof-sFRP-1* is unaffected by this treatment. **J** *Hof-sFRP-1* and *Hof-fz-1* (a posterior marker) were expressed in the ectopic mouths and tails when regenerating fragments are exposed to β-catenin RNAi (**K**) Inhibition of the *APC* gene by RNAi, causes the development of tail-like structures in the anterior and the midbody regions of a regenerating animal, as revealed by the expression/label of the posterior marker *fz-1gene.* All functional experiments shown in I-K are shown in [56]. **L-N** Expression domains, and the corresponding diagrams/colors, of the three Hox genes expressed in the hatchlings of the species *S. roscoffensis. SrHox1*, *SrHox5* and *SrHoxPost* denote the Hox paralogous groups to which each gene belongs. As in other bilaterian animals the expression domains of these genes are staggered along the major (A-P) body axis (figures taken from [41], with permission). **O** Scheme showing the expression domains of several bHLH genes in hatchlings of the species *S. roscoffensis*. The expression domains in early embryos (**P**, **R**) and hatchlings (**Q**, **S**) of some of these bHLH genes are shown. They contribute to the specification of neural structures (they overlap the domains of a pan-neuronal marker such as synaptotagmin; not shown). Panels O-S are taken, with permission, from Perea-Atienza et al. [45]

In the following sections we tackle the problem of subdividing the major body axis of the acoels in different domains, from the early specification of the Antero-Posterior and Dorso-Ventral axis to the final control of the region-specific patterns of neural differentiation (Fig. 5 for more details).

### Specifying the acoel major body *axis*

Specification of the major body axes (anteroposterior, AP, and dorsoventral, DV) in bilaterians is controlled by conserved signaling mechanisms. Many of these genetic programs and signaling pathways are established well before overt morphological bilateral symmetry is recognized in adults. The Wnt and bone morphogenetic protein (BMP) pathways are the primary regulators of AP and DV patterning, respectively (see e.g., [43]). Moreover, BMP and Wnt family members interact—with synergistic or antagonistic effects—with other signaling pathways, including the fibroblast growth factor (FGF), Notch, and Sonic Hedgehog (Shh) pathways, thereby regulating neural proliferation and differentiation [31]. We find both BMP-BMP antagonists and Wnt-Wnt antagonists being part of the mechanism controlling the two cardinal body axes in acoels (Fig. 5A-C, H–K).

All gene expression domains in this figure were obtained by colorimetric in situ hybridization methods.

Members of the Wnt/Wingless family were initially characterized as short-range inducers and long-range organizers of axis formation and organogenesis in bilaterians. Moreover, analysis of Wnt genes during gastrulation of the sea anemone *Nematostella* indicated that their roles in axial differentiation are ancient, predating the origin of Bilateria (reviewed in [28]). In fact, the presence of Wnt signaling is a novelty of metazoan animals [44]. In Acoela, several Wnt family members have been recently described, some of which possess divergent sequences [19, 56].

Though the embryonic axis formation in most bilaterian groups depends on the coordinated activities of these two signaling systems, in acoels we don’t have, so far, any functional evidence for the specific role of BMPs or Wnts in setting up these axes during early embryogenesis. However, the presence of members of these pathways (agonists and antagonists of BMPs and Wnts) has be revealed, by in situ hybridization, in late embryonic stages of the acoel species *H. miamia*. In the so-called “pill stage” (about 100 hpf; a pre-hatchling stage), Kimura and collaborators [34] show that the anterior, Wnt-pathway regulated, *sfrp-1,* and the posterior marker *fz-1* occupy opposite territories during this stage, suggesting that the AP axis has been established by then (see Fig. S5A, B in [34]). Similarly, *admp* and *bmp* (specifiers of the DV axis) are also detectable at opposite sides of the pill stage embryo, thus indicating the presence as well of a clear DV axis (see, also, Fig. S5A, B in [34]). Since earlier stages have not been analyzed we don’t know when the pathways are activated. In this context, further knockdown (or pharmacological) experiments should prove whether these genes are also functionally relevant in acoel embryo axis formation.

Despite the limited understanding of the functional role of Wnt and BMP pathways in embryogenesis, the involvement of these regulatory molecules in axis specification was investigated, instead and thoroughly, in the adults by Srivastava and colleagues [56], in which a series of experiments carried out in the regenerating *H. miamia* clearly established the role of this pathway in establishing the AP axis. Specifically, ectopic anterior or posterior structures developed in these acoel worms depending on whether they were treated with RNAi for positive or negative pathway regulators (Fig. 5I-K). While Wnt proteins regulate posterior identity (see also: e.g., [37]), how they mediate their effects is not completely understood in many animal groups, including Acoela. However, we do know that Wnt signaling is involved in maintenance of the AP axis during both tissue turnover and AP regeneration. With this observation in mind, Tewari and colleagues [61] used *β-catenin-1* RNAi and differential expression analysis to analyze the transcriptional effects of Wnt pathway inhibition or activation on AP axis formation in regenerating *H. miamia*. They demonstrated that inhibition of Wnt signaling during posterior regeneration led to the downregulation of a Hox gene (among several other transcription factors), pointing to a conserved regulatory link between Wnt pathway activity and Hox gene expression [6]. The molecular mechanisms controlling posterior regeneration were further analyzed by Ramirez et al. [51] who performed a transcriptomic analysis on regenerating *H. miamia*. In this study, Wnt-3 was described as an early response to wound healing, modeled by a head fragment with a posterior-facing blastema. Wnt-3 expression was localized to stem cells (*piwi-1*^+^ neoblasts), where it was regulated by Wnt-1. Moreover, Wnt-3 RNAi animals showed clear defects in axial specification, where head fragments failed to form posterior tissues and tail fragments failed to regenerate a visible blastema or mouth. Taken together, these studies emphasize that the AP axis in bilaterians is regulated by the (early) activities of the Wnt signaling pathway. However, the studies performed in Acoela did not analyze the specific effects of Wnt family members or their antagonists on neurogenesis.

With respect to the regulation of DV axis patterning, members of the transforming growth factor-beta family (e.g., dpp in *Drosophila* and its vertebrate homologs, the BMPs) and their extracellular antagonists (e.g., Chordin) are crucial in all bilaterians [27]. In fact, in most bilaterians, Bmp2/4 and Chordin form opposing expression gradients that control a downstream regulatory network that specifies the identities of different tissues along the DV axis (Fig. 5B, C). More recent work has shown that the divergent BMP family member Admp also participates in embryonic DV patterning. Specifically, *admp* and *bmp4* form complementary expression domains in both protostomes and deuterostomes [20]. Moreover, in Acoela, BMP signaling pathway components are also deployed along the DV axis,the roles of several of these factors have been analyzed more recently with knockdown technologies [38, 56].

### Localizing the brain and the nerve chords within the body

Downstream of the BMPs and their antagonists (ADMP, Chordin, and noggin), NS patterning along the DV axis in many (but not all) bilaterians is associated with the staggered expression of several transcription factors, such as nkx2.1/nkx2.2, nkx6, pax6, pax3/7, and msx (see examples in Fig. 5D-G). These transcription factors control the specification of neuronal cell types within their different domains (see: [13, 38]. While this regulatory network is well-understood in some bilaterian models (e.g., *Drosophila* and vertebrates), it remains to be characterized in many other groups, including Acoela.

To understand how a NS is positioned along the DV axis in Acoela, a recent study by Martin-Duran and colleagues [38] identified the expression patterns of relevant BMP/antagonist-regulated genes in *I. pulchra*. Interestingly, while the expression of BMP and ADMP was similar to those observed in other bilaterians, the active downstream DV transcription factors did not show clear staggered expression (Fig. 5B-G). Moreover, BMP signaling did not affect CNS development, indicating that the involvement of BMP/antagonist signaling in neurogenesis and DV nerve chord pattering evolved later in the remaining bilaterian groups (if acoels are basal bilaterians), or that the role of BMP/antagonists was secondarily lost in acoels (if they are extant members of the Ambulacraria). Notably, however, in a different species, *H. miamia*, inhibition of BMP or the ADMPs resulted in a mutual cross-regulatory interaction [56]. However, neither the activity of downstream genes nor the resulting effects on neurogenesis were evaluated in this study.

### Regionalizing the nervous system

Thus far, we have analyzed the specification of the two primary orthogonal axes in Acoela. We will now describe how these initial patterns regulate neurogenesis and the partition of the NS in subdomains, whether they are structural or functional.

The broad subdivisions of the neural ectoderm can be revealed by the study of expression domains of different transcription factors. The expression of some of these early genes can be detected in acoel species in the domains in which the CNS arises, and many of them patterned the NS in pre-bilaterian (cnidarian) nerve-nets [17]. Some of the examined examples have been studied in postembryonic development, mostly in late embryos, hatchlings, and juveniles: the Cdx (orthologue of Drosophila caudal), Otp (orthopedia), NK2.1, SoxB1, Evx, and Hox genes in *Convolutriloba longifissura* [24, 25], SoxB1, the Hox genes, and a variety of bHLH (basic helix-loop-helix genes in *S. roscoffensis* ([41, 45, 55]; see also Fig. 5L-S); and the POU genes Brn-1 and Brn-3 in *N. fusca* [50]. These genes seem to delimit areas of the NS, in many cases a bilobulate domain in the anterior ectoderm, the brain. However, in the absence of more detailed analysis of those domains and the absence of functional assays, the roles of most of the genes involved in the development of neural tissue are not resolved.

In a few cases, the roles of regulatory genes in NS formation have been analyzed in the context of adult NS regeneration. These analyses open the possibility of describing gene activities and their mutual interactions, which are known as gene regulatory networks or GRNs [46], that lead to the patterning of specific neural domains. One regulatory gene, the posterior Hox gene of the acoel *I. pulchra*, called IpHoxPost, has been knocked down and the phenotypic consequences have been analyzed [42]. In this study, treatment of posterior regenerating fragments with IpHoxPost RNAi knocked down the expression of IpHoxPost. This knockdown resulted in extensive changes in the morphology of the body, especially in the posterior half, where the nervous fibers were slightly reduced, an effect probably due to a decrease in the number of neural cells maintained or produced after the treatment. The area affected also showed defects in the deployment of mesodermal structures from the mouth to the copulatory organs. In this pioneering study, RNAi has demonstrated for the first time that the development of the posterior nervous tissue in *I. pulchra* is under the control of the posterior Hox gene [42], as it occurs in other bilaterians [67].

More recently, the patterning of the NS has been reanalyzed in the context of adult NS regeneration [30]. These authors have used single-cell sequencing methods to trace the development of postembryonic mature cell types from the stem cell-like (neoblast) populations. Interestingly, one subcluster of neoblasts, expressing the gene Sox1 (*sox-1*^+^ cells), seems to be dedicated to the production of neuronal types. Other transcription factors, such as the Vax and Nkx2-1 homologs, contributed to the specification of neural lineages, all of which were derived from the population of dedicated neoblast cells, as confirmed by the Vax and Nkx2-1 RNAi phenotypes. The study, although preliminary, is a significant attempt at identifying putative regulators of neuronal differentiation trajectories.

While the data are informative and tell us about the early specification of neuronal domains (e.g., the brain), we lack the proper functional analysis tools necessary to determine the specific functions of genes in development. The recent development of CRISPR (Clustered Regularly Interspaced Short Palindromic Repeats-based) and transgenic technologies in *H. miamia* should allow a better dissection of the process of neurogenesis in this, and potentially other, acoel systems [54].

Downstream of these regulatory systems, the expression levels of various effector genes, such as those encoding for neurotransmitter synthesis enzymes, are localized in specific subdomains of the NS [29], thus revealing the species-specific complexity in which neural cells or circuits are developmentally assembled [1, 23]. These patterned circuits will generate the full repertoire of behaviors that the acoels show in the laboratory and in the wild [18, 58, 66].

Other aspects of the acoel neurogenesis haven’t been studied yet, such as those controlling the pathfinding of neurons and the integration into functional circuits. These should become the necessary focus of attention in future research.

Moreover, the evolutionary history of the neural architectures, and their impressive diversity, within the phylum Xenacoelomorpha needs to be tackled with molecular techniques, so far available (as shown in this section) in very few species.

## Discussion: Future directions in the acoel nervous systems research

One of the most salient aspects of acoel flatworm nervous systems (NSs) is the architectural variability. While some older clades mostly contain anterior neural bundles of processes associated with a few cell bodies located preferentially in the statocyst area, others contain developed neuronal (brains) aggregates with an external layer of cell bodies and an internal dense neuropil. These latter brains have some peculiarities, such that they are infiltrated by other cell types (e.g., glandular, or muscular cells). In addition, the presence of track condensations in the form of nerve chords is a characteristic of most groups but not all. The number of cords is also variable, with a circumferential distribution, which is approximately symmetrical with respect to the dorsoventral plane.

Most of the research on characterizing the structure of the NS has been done either using conventional histology staining or, more recently, cross-reacting antibodies (raised against neurotransmitters), primarily in adult specimens. The results are still fragmented and have been primarily focused on the appearance of the “scaffold” of the NS. Immunochemical reactions against neurotransmitters only reveal where these are being synthesized/released. What are we missing if our primary research aim is a comprehensive understanding of acoel NS and its evolutionary trends? Some hints have already been presented in this paper but must be revisited here. They suggest avenues for future endeavors in the field. Let us move from the macroscopic to the molecular aspects.

- 1- Regarding the general architecture of the acoel NS, the field needs to understand better how neurons and associated cells are distributed (and connected) in the brain and nerve chords. This project should involve serial TEM (or any of its modern, semiautomatic substitutes, e.g., FIB, Focused Ion Beam). The main aim is to have a complete representation of neurons in the NS, irrespective of the neurotransmitters they use. Moreover, having access to “all” neurons, plus associated cells, should be immensely informative, giving us an unprecedented resolution on the cellular structure and connectivity within NSs. The taxonomic range should be a principle that guides us in selecting species, particularly those species that diversified at the deepest nodes (*Diophistoporida*, *Parotomellida*, and *Hofsteniedae*). Other, more advanced Acoels, with elaborated bilobate brains, should also be incorporated following, for example, what has been performed for *S. roscoffensis* [4],our current work). Moreover, the incorporation of new AI tools to the reconstruction of connectomic maps across species should help understanding how the variability of neural architectures occurs in their finest details (e.g. [40], Biorxiv).

In this context it is important to re-emphasize here that most analysis of acoel neuroanatomy have been done using a limited set of neurotransmitter antibodies (mostly serotonin), thus, assumptions about the evolution of the nervous system should not be boldly drawn from a few antibodies staining against common neurotransmitters, as differences between taxa could, also, reflect adaptations to different lifestyles and correlated body forms. Applying these (EM-based) imaging methods used in *Symsagittifera* might substantially alter our evolutionary scenarios.

- 2- A detailed map of neurotransmitter expression should accompany a comprehensive map of cells in the brain of some acoels. This could be accomplished systematically by combining high-resolution immunochemistry/confocal microscopy with TEM maps. Other technologies, such as spatial transcriptomics (now reaching cellular resolution), should help build such a map.
- 3- The study of the embryology of the acoel NS is a field that needs to be addressed. Only a few attempts [26], for *Neochildia* and, more recently, Kimura et al. [34] and [35], for *Hofstenia*) have been made. More detailed maps should be developed via cell ablation and transplantation experiments. We still need a comprehensive analysis of the neurogenic lineage and determine how neighbors regulate the specified neurons through signaling mechanisms. The variability of early embryogenesis, as exemplified by new stages, such as the dimple in *Hofstenia*, prompts us to suggest further analysis of early embryogenesis events in different clades. How much variability is there in early embryogenesis? What are the specificities (and commonalities) of NS development across the Acoela?

Moreover, knockdown methodologies should be developed and applied to the study of early embryos. The regulatory mechanisms involved in setting the embryonic axis or the specification of neural phenotypes are still missing the incorporation of functional analysis.

- 4- What is the compositional variability of the NS in different acoels? How many cell types exist in the NS (or in the animal)? This should be addressed by higher-resolution single-cell sequencing. As difficult as it is, single-cell methodologies should improve where cell types can be unambiguously identified. The study of cell types in adults can be extended to other periods of development, allowing us to trace specific developmental trajectories for different cell types. In addition, to be fully informative, cell clusters identified in single-cell sequencing data should be mapped in the organism body. Knowing which cell types we have and where they are located should provide us with a unique opportunity to understand the finer details of the NS cellular architecture. This objective is complementary to that suggested in (1).
- 5- How do gene regulatory networks control the formation of the NS? This is a fundamental, though challenging, endeavor. Ideally, we would like to trace all the regulatory interactions that control neurogenesis, from establishing early neuroblasts to their final, differentiated phenotype. Transcription factor interactions guide the process but do it in a complex array of interactions. Confirming them is the real challenge. Currently, a combination of genomic methods (ATAC, Chip seq, comparative transcriptomics, etc.) is helping many researchers (in other areas and systems) trace regulatory interactions occurring at different times in development and different areas of the embryo/juvenile/adult. These interactions should be “probed” if knockdown techniques are incorporated.
- 6- The construction of NSs, with their reproducible and exquisite wiring pattern, suggests the need to pay further attention to those aspects that regulate cellular and process migration, branching, and target selection. Thus far, in the Acoela, we have very little information on how these processes occur and where, when, and how they are regulated. Particular attention should be paid to deciphering these mechanisms at the cellular and molecular levels.

All the above represent a series of putative avenues for acoel NS research and are aspects that are neglected and need to be addressed. Through a more comprehensive analysis of the different levels of NS control, we will be able to understand the evolutionary paths that the NS has taken in this fascinating group of animals. A better understanding of the general morphology and cellular composition of the different acoel NSs will allow us to start tackling new functional aspects, such as studying acoel behavior or their interaction with the environment.

## Data Availability

Doesn’t apply.
